# Synthesis and crystal structure of 4-(2-ammonio­eth­yl)morpholin-4-ium di­chlorido­diiodido­cadmate/chlorido­tri­iodido­cadmate (0.90/0.10)

**DOI:** 10.1107/S2056989016013967

**Published:** 2016-09-05

**Authors:** Najla Mahbouli Rhouma, Ali Rayes, Francesco Mezzadri, Gianluca Calestani, Mohamed Loukil

**Affiliations:** a Laboratoire des Sciences des Matériaux et d’Environnement, Faculté des Sciences, Université de Sfax, BP 1171, Route de Soukra, 3018 Sfax, Tunisia; b Unité de Recherche, Catalyse et Matériaux pour l’Environnement et les Procédés, URCMEP, (UR11ES85), Faculté des Sciences de Gabès, Campus Universitaire, 6072 Gabès, Tunisia; c Dipartimento di Chimica, Universitá di Parma, Parco Area delle Scienze 17A, I-43124 Parma, Italy

**Keywords:** crystal structure, organic inorganic hybrid materials, hydrogen bonds

## Abstract

The crystal structure of the title compound, (C_6_H_16_N_2_O)[CdCl_1.90_I_2.10_], a new organic–inorganic hybrid salt synthesized in the form of single crystals, consists of discrete statistically distributed di­chlorido­diiodido­cadmate/chlorido­tri­iodido­cadmate anions (occupancy ratio 0.90:0.10) and 4-(2-ammonio­eth­yl)morpholin-4-ium cations, [NH_3_(CH_2_)_2_NH(CH_2_)_4_O]^2+^. The cations are linked by inter­molecular N—H⋯O hydrogen bonds, forming corrugated chains extending parallel to the *c* axis. The [CdCl_1.90_I_2.10_]^2−^ tetra­halidocadmate anions lie between the chains to maximize the electrostatic inter­actions and are connected with the organic cations *via* N—H⋯Cl and C—H⋯Cl(I) hydrogen bonds developing in the *ab* plane and leading to the formation of a three-dimensional network structure. The tetra­coordinate Cd^II^ atom has a distorted tetra­hedral conformation, with a τ_4_ index of 0.87.

## Abstract 2

The crystal structure of a new inorganic–organic hybrid material, (C_6_H_16_N_2_O)[CdCl_1.90_I_2.10_], consists of 4-(2-ammonio­eth­yl)morpholin-4-ium cations and di­chlorido­diiodido­cadmate/chlorido­tri­iodido­cadmate (0.90/0.10) anions connected by hydrogen bonds into a three-dimensional network.

## Chemical context   

Inorganic–organic hybrid materials are crystalline materials in which the organic and inorganic moieties are connected *via* covalent, ionic or hydrogen bonds inside the structures. These materials provide the opportunity to combine intended properties of both the organic and inorganic components when they are self-assembled in the solid state. For instance, inorganic metal halides may be associated with functionalized organic mol­ecules (carb­oxy­lic acids, amides or amines) to produce two different types of hybrid materials, both of which are of technological inter­est. When the organic mol­ecules coordinate to the metal ions of the metal halides, the resulting products are called coordination polymers or coordination compounds. The coordination polymers may be related to compounds with metal–organic framework (MOF) structures. These MOF materials have been studied intensively due to their intriguing structures and their potentially inter­esting properties, including high porosity, structural flexibility, nonlinear optical behaviour or magnetic properties (Mitzi *et al.*, 2001 ▸).

Once the moieties are combined as perhalidometalate anions and organic cations, the resulting products are called ionic organic–inorganic hybrid materials. These materials frequently conserve the properties of the individual parts, *i.e.* the organic component may add structural diversity and optical properties (fluorescence and luminescence), while the inorganic component potentially contributes to mechanical resistance, thermal stability, electric properties (conductor, semiconductor, insulator) or magnetic properties (Ciurtin *et al.*, 2001 ▸). Well-tested applications of these ionic hybrids include light-emitting diodes (LEDs) (Ciurtin *et al.*, 2001 ▸). Moreover, in these materials, the crystal packing is ensured by Coulombic inter­actions and hydrogen bonds. These non-covalent weak forces of N—H⋯halide–metal play a vital role in supra­molecular chemistry and continue to attract much attention. As a contribution to the investigation of the above materials, we report here the crystal structure of one such compound, (C_6_H_16_N_2_O)[CdCl_1.90_I_2.10_], formed from the reaction of 4-(2-amino­eth­yl)morpholine and cadmium iodide in hydro­chloric acid.
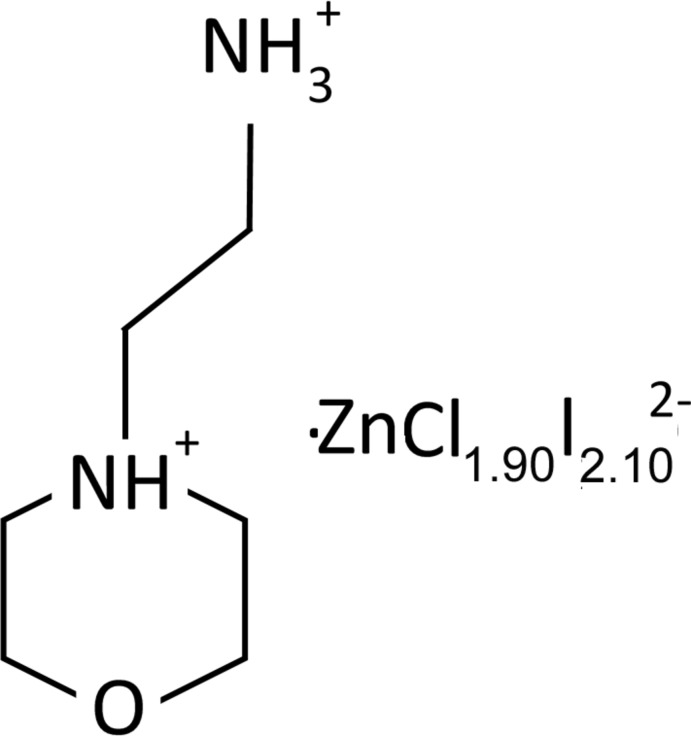


## Structural commentary   

**Figure 1:**
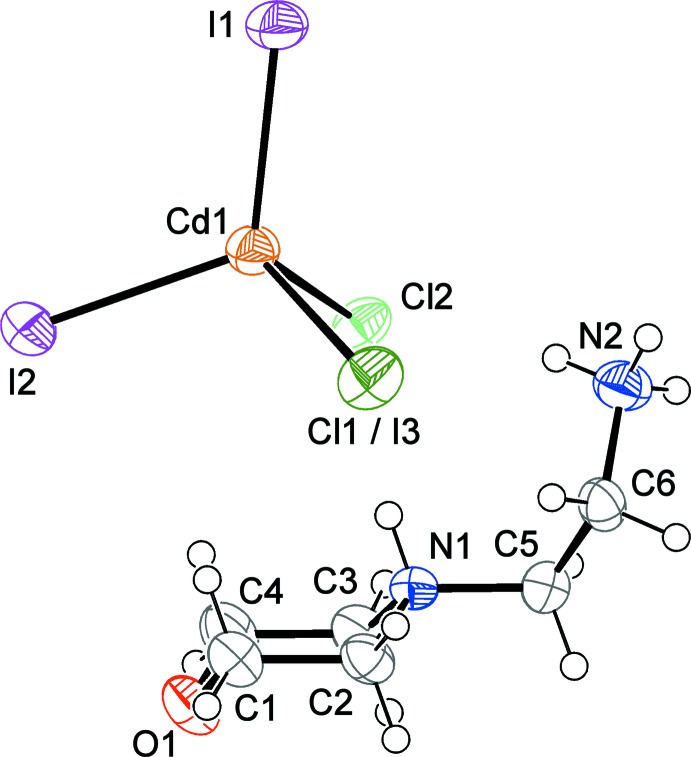
The asymmetric unit of the title compound, with displacement ellipsoids drawn at the 50% probability level.

The asymmetric unit of the title hybrid salt, (C_6_H_16_N_2_O)[CdCl_1.90_I_2.10_], contains one [NH_3_(CH_2_)_2_NH(CH_2_)_4_O]^2+^ cation and one tetra­halidocadmate anion with average composition [CdCl_1.90_I_2.10_]^2−^ (Fig. 1 ▸), both occupying general positions in the unit cell. Each Cd^II^ atom is tetra­coordinate in a distorted tetra­hedral environment defined by two Cl atoms and two I atoms in 90% of the cases and by one Cl atom and three I atoms in the remaining 10%. The disorder involves only one halogen site and implicates the statistical presence of the Cl1 and I3 atoms. The partial presence of iodine in this site reflects a small increase of the Cd—Cl1 bond length when compared with Cd—Cl2 [2.5919 (11) and 2.5148 (11) Å, respectively]. The other Cd—Cl and Cd—I bond lengths are in agreement with the values reported in the literature (Sato *et al.*, 1986 ▸; Ishihara *et al.*, 2000 ▸). The average distortion of the [CdCl_1.90_I_2.10_]^2−^ anion from the ideal tetra­hedral conformation can be confirmed by the values of the two largest angles around the Cd^II^ atom [115.28 (2) and 120.96 (4)°]. These two angles can also be used to calculate the τ_4_ structural parameter introduced by Yang *et al.* (2007 ▸) for complexes with coordination number four (CN = 4) to qu­antify this distortion. This parameter is defined as τ_4_ = [360 − (α + β)]/(360 − 2θ), where α and β are the two greatest valence angles around the central atom and θ = 109.5° is the ideal tetra­hedral angle. τ_4_ can range from 1 to 0, passing from an ideal tetra­hedral to a perfect square-planar conformation. The τ_4_ value of the present structure is 0.87, indicative of a distorted tetra­hedral environment. The bond angles involving the Cd^II^ atom range between 94.15 (3) and 120.95 (4)°. The lower value, significantly smaller than all the other bond angles, is observed for the Cl1—Cd—Cl2 angle. This distortion is too large to be attributed uniquely to the structural disorder involving the Cl1 site and suggests the involvement of the Cl atoms in a complex system of N—H⋯Cl hydrogen bonds as being responsible of the phenomenon.

In the organic entity, the morpholine ring adopts a typical chair confirmation and all the geometrical features agree with those found in 4-(2-ammonio­eth­yl)morpholin-4-ium tetra­chlorido­zincate (El Glaoui *et al.*, 2008 ▸; Lamshöft *et al.*, 2011 ▸).

## Supra­molecular features   

**Figure 2:**
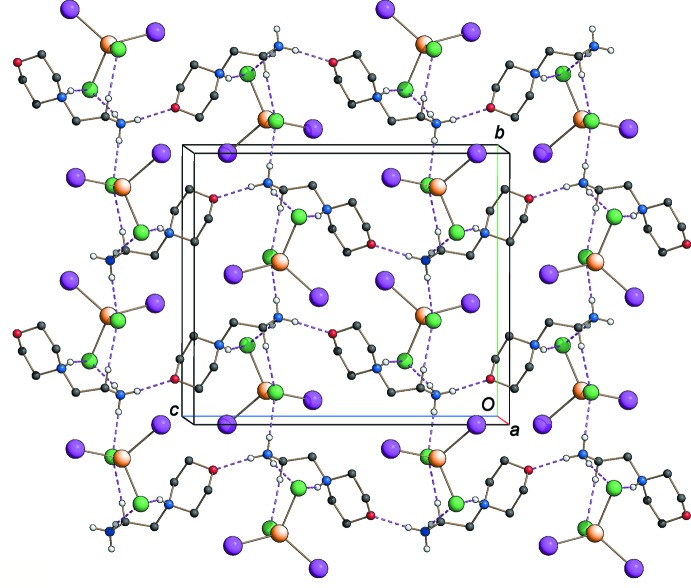
Packing diagram of the title compound viewed approximately along the *a* axis, showing the three-dimensional hydrogen-bonding network (dashed lines). Only the hydrogen bonds formed when the disordered halogen site is occupied by the Cl atom (*i.e.* the predominant situation) are reported for clarity.

As depicted in Fig. 1 ▸, the organic entity is double protonated at both the N atoms (N1 and N2) to ensure charge balance. In connectivity terms, the cations are linked by inter­molecular N—H⋯O hydrogen bonds involving one of the ammonium H atoms, leading to a *C*(6) chain motif, with the corrugated chains extending parallel to the *c* axis. The [CdCl_1.90_I_2.10_]^2−^ anions lie between the chains to maximize the electrostatic inter­actions and are connected with the organic cations *via* N—H⋯Cl and C—H⋯Cl1(I3) hydrogen bonds (Table 1 ▸). These hydrogen bonds develop in the *ab* plane, leading to the formation of a three-dimensional network structure (Fig. 2 ▸). The analysis of the N—H⋯Cl distances, varying between 2.38 and 2.40 Å, shows that they are much shorter than the sum of the van der Waals radii, indicating a rather strong character of these hydrogen bonds.

## Database survey   

A search of the Cambridge Structural Database (Version 5.37; last update February 2016; Groom *et al.*, 2016 ▸) for related compounds showed the appearance of the zinc analogue of formula (C_6_H_16_N_2_O)[ZnCl_4_] (El Glaoui *et al.*, 2008 ▸; Lamshöft *et al.*, 2011 ▸), in which the Zn^II^ atom is coordinated by four Cl atoms in a slightly distorted tetra­hedral environment (τ_4_ = 0.93). In spite of a common symmetry and of a certain similitude in the unit-cell parameters, this and the title compound are not isotypic. Due to a major efficency in the hydrogen-bond formation, the [ZnCl_4_]^2−^ anions inter­act in a different way with the cations, building layers parallel to the *ac* plane and not, as in the title compound, a three-dimensional network structure. Calculation of the index geometry for four-coordinated atoms, τ_4_, shows that the distortion of the tetra­halidocadmate unit in the present compound (τ_4_ = 0.87) is not only larger than that observed in the previously mentioned [ZnCl_4_]^2−^ analogue, but also than the one of the [ZnI_2_Cl_2_]^2−^ unit (τ_4_ = 0.95) in the salt with *N*-methyl-1,3,5-tri­aza-7-phospha­adamantane (Smolenski *et al.*, 2009 ▸). This confirms the involvement of the Cl atoms in a complex system of strong N—H⋯Cl hydrogen bonds at the origin of tetra­hedral distortion observed in the present case.

## Synthesis and crystallization   

Crystals of (C_6_H_16_N_2_O)[CdCl_1.90_I_2.10_] were prepared starting from CdI_2_ (purity 99%, Sigma–Aldrich), 4-(2-aminioeth­yl)morpholine (purity 99%, Sigma–Aldrich) and HCl (37% *w*/*w*), weighted in stoichiometric amounts conforming to the idealized equation:

NH_2_(CH_2_)_2_N(CH_2_)_4_O + CdI_2_ + 2HCl → [NH_3_(CH_2_)_2_NH(CH_2_)_4_O]CdCl_2_I_2_.

An aqueous solution of 4-(2-amino­eth­yl)morpholine was added dropwise to a mixture of CdI_2_ and HCl in a minimum amount of water (20 ml). After stirring for a period of 4 h, the resulting solution was placed in a Petri dish and allowed to evaporate slowly at room temperature. Single crystals of the title compound, suitable for X-ray diffraction analysis, were obtained after several days (yield ∼78%).

## Refinement   

Crystal data, data collection and structure refinement details are summarized in Table 2 ▸. One halogen site was found to be statistically occupied by Cl and I atoms (Cl1 and I3). The site-occupancy factors were refined by assuming full site occupancy and by using the same coordinates and anisotropic displacement parameters for both atoms. The N-bound morpholinium H atom was located in a difference Fourier map and refined freely. All other H were placed geometrically and refined as riding, with N—H = 0.89 Å and C—H = 0.97 Å. The isotropic displacement parameters of the ammonium H atoms were refined freely, whereas the remaining ones were refined with *U*_iso_(H) = 1.2*U*_eq_(C). A rotating model was used for the ammonium group.

## Supplementary Material

Crystal structure: contains datablock(s) I. DOI: 10.1107/S2056989016013967/wm5315sup1.cif

Structure factors: contains datablock(s) I. DOI: 10.1107/S2056989016013967/wm5315Isup2.hkl

CCDC reference: 1501997

Additional supporting information: crystallographic information; 3D view; checkCIF report

## Figures and Tables

**Table 1:** Hydrogen-bond geometry (Å, °)

*D*—H⋯*A*	*D*—H	H⋯*A*	*D*⋯*A*	*D*—H⋯*A*
N2—H2N⋯O1^i^	0.89	2.01	2.894 (4)	172
N2—H3N⋯Cl2	0.89	2.40	3.279 (4)	168
N2—H4N⋯Cl1^ii^	0.89	2.39	3.221 (4)	156
C2—H2*A*⋯I3	0.97	2.98	3.637 (4)	126
C6—H6*A*⋯Cl1	0.97	2.73	3.577 (4)	146
C6—H6*A*⋯I3	0.97	2.73	3.577 (4)	146
N1—H1N⋯Cl2	0.89 (5)	2.38 (5)	3.180 (3)	149 (4)

Symmetry codes: (i) 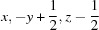; (ii) 
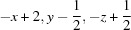
.

**Table 2:** Experimental details

Crystal data	
Chemical formula	(C_6_H_16_N_2_O2)[CdCl_1.90_I_2.10_]
*M*_r_	578.68
Crystal system, space group	Monoclinic, *P*2_1_/*c*
Temperature (K)	294
*a*, *b*, *c* (Å)	6.7773 (14), 13.870 (3), 16.104 (3)
β (°)	93.788 (3)
*V* (Å^3^)	1510.5 (5)
*Z*	4
Radiation type	Mo *K*α
μ (mm^−1^)	6.06
Crystal size (mm)	0.37 × 0.22 × 0.20

Data collection	
Diffractometer	Bruker SMART CCD
Absorption correction	Multi-scan (*SADABS*; Bruker, 2008 ▸)
*T*_min_, *T*_max_	0.218, 0.415
No. of measured, independent and observed [*I* > 2σ(*I*)] reflections	16802, 2879, 2626
*R*_int_	0.033
(sin θ/λ)_max_ (Å^−1^)	0.611

Refinement	
*R*[*F* ^2^ > 2σ(*F* ^2^)], *wR*(*F* ^2^), *S*	0.024, 0.055, 1.09
No. of reflections	2879
No. of parameters	136
H-atom treatment	H atoms treated by a mixture of independent and constrained refinement
Δρ_max_, Δρ_min_ (e Å^−3^)	1.09, −0.82

Computer programs: *APEX2* and *SAINT* (Bruker, 2008 ▸), *SHELXT* (Sheldrick, 2015*a* ▸), *SHELXL2014* (Sheldrick, 2015*b* ▸), *ORTEP-3* (Farrugia, 2012 ▸) and *SCHAKAL* (Keller, 1999 ▸).

## supplementary crystallographic information

### Crystal data

**Table:**

(C_6_H_16_N_2_O2)[CdCl_1.90_I_2.10_]	*F*(000) = 1063
*M**_r_* = 578.68	*D*_x_ = 2.545 Mg m^−^^3^
Monoclinic, *P*2_1_/*c*	Mo *K*α radiation, λ = 0.71073 Å
*a* = 6.7773 (14) Å	Cell parameters from 386 reflections
*b* = 13.870 (3) Å	θ = 8.5–19.7°
*c* = 16.104 (3) Å	µ = 6.06 mm^−^^1^
β = 93.788 (3)°	*T* = 294 K
*V* = 1510.5 (5) Å^3^	Prism, colourless
*Z* = 4	0.37 × 0.22 × 0.20 mm

### Data collection

**Table:**

Bruker SMART CCD diffractometer	2626 reflections with *I* > 2σ(*I*)
ω scan	*R*_int_ = 0.033
Absorption correction: multi-scan (*SADABS*; Bruker, 2008)	θ_max_ = 25.7°, θ_min_ = 1.9°
*T*_min_ = 0.218, *T*_max_ = 0.415	*h* = −8→8
16802 measured reflections	*k* = −16→16
2879 independent reflections	*l* = −19→19

### Refinement

**Table:**

Refinement on *F*^2^	0 restraints
Least-squares matrix: full	Hydrogen site location: mixed
*R*[*F*^2^ > 2σ(*F*^2^)] = 0.024	H atoms treated by a mixture of independent and constrained refinement
*wR*(*F*^2^) = 0.055	*w* = 1/[σ^2^(*F*_o_^2^) + (0.0208*P*)^2^ + 2.0157*P*] where *P* = (*F*_o_^2^ + 2*F*_c_^2^)/3
*S* = 1.09	(Δ/σ)_max_ < 0.001
2879 reflections	Δρ_max_ = 1.09 e Å^−^^3^
136 parameters	Δρ_min_ = −0.82 e Å^−^^3^

### Special details

**Table:**

Geometry. All esds (except the esd in the dihedral angle between two l.s. planes) are estimated using the full covariance matrix. The cell esds are taken into account individually in the estimation of esds in distances, angles and torsion angles; correlations between esds in cell parameters are only used when they are defined by crystal symmetry. An approximate (isotropic) treatment of cell esds is used for estimating esds involving l.s. planes.

### Fractional atomic coordinates and isotropic or equivalent isotropic displacement parameters (Å^2^)

**Table:**

	*x*	*y*	*z*	*U*_iso_*/*U*_eq_	Occ. (<1)
Cd1	0.58963 (4)	0.38807 (2)	0.25555 (2)	0.03751 (9)	
I1	0.42207 (4)	0.42832 (2)	0.10184 (2)	0.04982 (10)	
I2	0.50580 (4)	0.51493 (2)	0.37657 (2)	0.05043 (10)	
Cl1	0.96790 (13)	0.38224 (6)	0.24065 (6)	0.0529 (4)	0.8977 (19)
I3	0.96790 (13)	0.38224 (6)	0.24065 (6)	0.0529 (4)	0.1023 (19)
Cl2	0.57453 (14)	0.22097 (7)	0.31540 (7)	0.0399 (2)	
O1	0.9564 (4)	0.3339 (2)	0.55915 (17)	0.0457 (7)	
N1	0.9882 (5)	0.2064 (2)	0.41993 (19)	0.0305 (7)	
N2	0.9358 (5)	0.1077 (3)	0.2309 (2)	0.0431 (9)	
H2N	0.9550	0.1249	0.1788	0.067 (16)*	
H3N	0.8266	0.1357	0.2470	0.059 (15)*	
H4N	0.9228	0.0439	0.2335	0.14 (3)*	
C1	1.0431 (6)	0.3661 (3)	0.4858 (3)	0.0417 (10)	
H1A	1.1421	0.4147	0.5005	0.050*	
H1B	0.9420	0.3953	0.4484	0.050*	
C2	1.1375 (6)	0.2838 (3)	0.4422 (2)	0.0364 (9)	
H2A	1.1931	0.3074	0.3921	0.044*	
H2B	1.2443	0.2572	0.4782	0.044*	
C3	0.8898 (6)	0.1772 (3)	0.4971 (3)	0.0423 (10)	
H3A	0.9854	0.1458	0.5357	0.051*	
H3B	0.7846	0.1316	0.4826	0.051*	
C4	0.8059 (7)	0.2645 (3)	0.5379 (3)	0.0478 (11)	
H4A	0.7052	0.2936	0.5003	0.057*	
H4B	0.7440	0.2449	0.5878	0.057*	
C5	1.0752 (6)	0.1214 (3)	0.3779 (3)	0.0391 (9)	
H5A	0.9875	0.0666	0.3823	0.047*	
H5B	1.2006	0.1052	0.4071	0.047*	
C6	1.1085 (6)	0.1385 (3)	0.2867 (2)	0.0362 (9)	
H6A	1.1331	0.2065	0.2780	0.043*	
H6B	1.2248	0.1031	0.2722	0.043*	
H1N	0.894 (7)	0.232 (3)	0.386 (3)	0.041 (12)*	

### Atomic displacement parameters (Å^2^)

**Table:**

	*U*^11^	*U*^22^	*U*^33^	*U*^12^	*U*^13^	*U*^23^
Cd1	0.03783 (17)	0.04165 (17)	0.03285 (16)	0.00247 (13)	0.00088 (12)	0.00227 (12)
I1	0.04754 (18)	0.0661 (2)	0.03486 (16)	0.01088 (14)	−0.00489 (12)	0.00216 (13)
I2	0.05227 (18)	0.0630 (2)	0.03538 (16)	0.01156 (14)	−0.00161 (13)	−0.00926 (13)
Cl1	0.0452 (6)	0.0470 (6)	0.0661 (7)	−0.0028 (4)	0.0006 (4)	0.0049 (4)
I3	0.0452 (6)	0.0470 (6)	0.0661 (7)	−0.0028 (4)	0.0006 (4)	0.0049 (4)
Cl2	0.0285 (5)	0.0382 (5)	0.0524 (6)	−0.0018 (4)	−0.0011 (4)	0.0063 (4)
O1	0.0485 (17)	0.0536 (18)	0.0356 (16)	−0.0092 (14)	0.0067 (13)	−0.0116 (14)
N1	0.0276 (16)	0.0342 (17)	0.0291 (16)	0.0006 (13)	−0.0026 (13)	−0.0022 (13)
N2	0.038 (2)	0.059 (3)	0.032 (2)	0.0007 (17)	0.0003 (15)	−0.0022 (17)
C1	0.045 (2)	0.043 (2)	0.037 (2)	−0.0047 (19)	0.0025 (19)	−0.0054 (18)
C2	0.032 (2)	0.043 (2)	0.034 (2)	−0.0068 (17)	0.0018 (16)	−0.0048 (17)
C3	0.044 (2)	0.047 (2)	0.037 (2)	−0.0093 (19)	0.0056 (18)	−0.0011 (19)
C4	0.043 (2)	0.060 (3)	0.042 (2)	−0.011 (2)	0.0130 (19)	−0.012 (2)
C5	0.041 (2)	0.035 (2)	0.041 (2)	0.0066 (18)	−0.0019 (18)	−0.0040 (17)
C6	0.031 (2)	0.038 (2)	0.040 (2)	−0.0025 (16)	0.0036 (17)	−0.0096 (18)

### Geometric parameters (Å, º)

**Table:**

Cd1—Cl2	2.5148 (11)	C1—H1A	0.9700
Cd1—Cl1	2.5919 (11)	C1—H1B	0.9700
Cd1—I1	2.7124 (6)	C2—H2A	0.9700
Cd1—I2	2.7135 (5)	C2—H2B	0.9700
O1—C1	1.425 (5)	C3—C4	1.507 (6)
O1—C4	1.428 (5)	C3—H3A	0.9700
N1—C5	1.499 (5)	C3—H3B	0.9700
N1—C2	1.503 (5)	C4—H4A	0.9700
N1—C3	1.505 (5)	C4—H4B	0.9700
N1—H1N	0.89 (5)	C5—C6	1.521 (6)
N2—C6	1.490 (5)	C5—H5A	0.9700
N2—H2N	0.8900	C5—H5B	0.9700
N2—H3N	0.8900	C6—H6A	0.9700
N2—H4N	0.8900	C6—H6B	0.9700
C1—C2	1.504 (6)		
			
Cl2—Cd1—Cl1	94.15 (3)	N1—C2—H2B	109.5
Cl2—Cd1—I1	120.95 (3)	C1—C2—H2B	109.5
Cl1—Cd1—I1	106.20 (3)	H2A—C2—H2B	108.1
Cl2—Cd1—I2	107.85 (3)	N1—C3—C4	110.1 (3)
Cl1—Cd1—I2	110.00 (2)	N1—C3—H3A	109.6
I1—Cd1—I2	115.280 (18)	C4—C3—H3A	109.6
C1—O1—C4	109.8 (3)	N1—C3—H3B	109.6
C5—N1—C2	113.0 (3)	C4—C3—H3B	109.6
C5—N1—C3	111.7 (3)	H3A—C3—H3B	108.2
C2—N1—C3	108.9 (3)	O1—C4—C3	111.3 (3)
C5—N1—H1N	108 (3)	O1—C4—H4A	109.4
C2—N1—H1N	108 (3)	C3—C4—H4A	109.4
C3—N1—H1N	106 (3)	O1—C4—H4B	109.4
C6—N2—H2N	109.5	C3—C4—H4B	109.4
C6—N2—H3N	109.5	H4A—C4—H4B	108.0
H2N—N2—H3N	109.5	N1—C5—C6	113.7 (3)
C6—N2—H4N	109.5	N1—C5—H5A	108.8
H2N—N2—H4N	109.5	C6—C5—H5A	108.8
H3N—N2—H4N	109.5	N1—C5—H5B	108.8
O1—C1—C2	111.2 (3)	C6—C5—H5B	108.8
O1—C1—H1A	109.4	H5A—C5—H5B	107.7
C2—C1—H1A	109.4	N2—C6—C5	112.2 (3)
O1—C1—H1B	109.4	N2—C6—H6A	109.2
C2—C1—H1B	109.4	C5—C6—H6A	109.2
H1A—C1—H1B	108.0	N2—C6—H6B	109.2
N1—C2—C1	110.7 (3)	C5—C6—H6B	109.2
N1—C2—H2A	109.5	H6A—C6—H6B	107.9
C1—C2—H2A	109.5		

### Hydrogen-bond geometry (Å, º)

**Table:**

*D*—H···*A*	*D*—H	H···*A*	*D*···*A*	*D*—H···*A*
N2—H2*N*···O1^i^	0.89	2.01	2.894 (4)	172
N2—H3*N*···Cl2	0.89	2.40	3.279 (4)	168
N2—H4*N*···Cl1^ii^	0.89	2.39	3.221 (4)	156
C2—H2*A*···I3	0.97	2.98	3.637 (4)	126
C6—H6*A*···Cl1	0.97	2.73	3.577 (4)	146
C6—H6*A*···I3	0.97	2.73	3.577 (4)	146
N1—H1*N*···Cl2	0.89 (5)	2.38 (5)	3.180 (3)	149 (4)

Symmetry codes: (i) *x*, −*y*+1/2, *z*−1/2; (ii) −*x*+2, *y*−1/2, −*z*+1/2.
